# Supplementary material for: A Likelihood Approach for Real-Time Calibration of Stochastic Compartmental Epidemic Models
Source: PLoS Comput Biol. 2017 Jan 17;13(1):e1005257. doi: 10.1371/journal.pcbi.1005257 (PMC5240920; doi:10.1371/journal.pcbi.1005257)
Supplement: S1 File — (TAR.GZ) [file pcbi.1005257.s014.tar.gz › HSPH_Online-SI-Revision/IPoi09/n1-severe/n1-severe_BENCH_table8.pdf]

1	R0	{1.51081, 13.7078}	{2.26331, 19.3932}	{{2.26161, 19.3849}, {2.26161, 19.3849}}	{49.8079, 41.4759}	inf	54.	63.7759	{25., 128.}	48.6271	0.183512	newly	28.	20.7931	{8., 34.}	45.197	0.275478	Reff	0.70857	0.424945	{0.303056, 0.545048}	43.5651	ar	656.	860.138	{836., 884.}	31.1186
2	R0	{1.73182, 11.3873}	{2.42153, 19.4445}	{{2.42205, 19.4475}, {2.42205, 19.4475}}	{39.8259, 70.756}	inf	39.	52.5424	{18., 103.}	70.5346	0.238645	newly	19.	16.8475	{5., 33.}	59.6789	0.316682	Reff	0.680605	0.382143	{0.256737, 0.481988}	48.6415	ar	653.	878.966	{845., 905.}	34.6043
3	R0	{1.50449, 10.3474}	{2.26279, 19.3779}	{{2.26161, 19.3849}, {2.26161, 19.3849}}	{50.4025, 87.2733}	inf	38.	88.9831	{42., 140.}	135.147	0.335662	newly	22.	29.6949	{11., 50.}	55.624	0.199157	Reff	0.664985	0.488468	{0.332457, 0.689791}	37.1026	ar	651.	861.136	{840., 884.}	32.2789
4	R0	{1.63493, 9.1955}	{2.81237, 19.8593}	{{2.7811, 19.6712}, {2.81635, 19.898}}	{72.0178, 115.968}	inf	17.	86.5538	{45., 147.}	409.14	0.656477	newly	9.	27.	{12., 46.}	200.342	0.414639	Reff	0.614734	0.319236	{0.225308, 0.394289}	53.6285	ar	663.	924.6	{907., 942.}	39.457
5	R0	{1.53822, 8.05553}	{2.72617, 19.7199}	{{2.66225, 19.6041}, {2.79269, 19.984}}	{77.229, 144.799}	inf	26.	68.6933	{38., 108.}	164.513	0.386164	newly	17.	21.4533	{12., 34.}	44.0784	0.158429	Reff	0.642976	0.294465	{0.218304, 0.365842}	54.2028	ar	621.	917.	{896., 935.}	47.6651
6	R0	{1.69374, 17.0793}	{2.13983, 19.51}	{{2.11476, 19.2116}, {2.1968, 19.9579}}	{26.3375, 14.2321}	inf	76.	76.1746	{37., 127.}	36.5288	0.176282	newly	35.	24.9841	{12., 40.}	37.6871	0.233657	Reff	0.753714	0.532599	{0.357148, 0.738125}	37.7243	ar	662.	831.	{794., 869.}	25.5287
7	R0	{1.42179, 11.8031}	{2.28158, 19.0521}	{{2.23915, 18.4504}, {2.30371, 19.6625}}	{60.4721, 61.4161}	inf	20.	43.7941	{18., 75.}	126.029	0.306012	newly	9.	14.2647	{4., 23.}	79.7386	0.244467	Reff	0.720848	0.385381	{0.292571, 0.4747}	46.7486	ar	517.	858.191	{825., 885.}	65.9944
8	R0	{1.46683, 6.95875}	{2.90774, 18.6643}	{{2.8568, 18.4249}, {2.96978, 18.9272}}	{98.2332, 168.214}	inf	0.001	48.2838	{25., 85.}	4.82828×10^{6}	2.63239	newly	0.1	16.0135	{7., 28.}	15913.5	2.14307	Reff	0.50899	0.229223	{0.177631, 0.282823}	54.9652	ar	653.	933.5	{917., 947.}	42.9556
9	R0	{1.34525, 11.505}	{2.14867, 19.4535}	{{2.11476, 19.2116}, {2.1968, 19.9579}}	{59.7229, 69.0875}	inf	36.	67.3651	{31., 127.}	95.7672	0.264754	newly	22.	23.5079	{9., 43.}	50.1443	0.247899	Reff	0.768138	0.503565	{0.335889, 0.722799}	42.1586	ar	532.	837.429	{798., 873.}	57.4114
10	R0	{1.77958, 14.372}	{2.42071, 19.4476}	{{2.42205, 19.4475}, {2.42205, 19.4475}}	{36.0271, 35.3159}	inf	31.	64.5085	{24., 134.}	119.245	0.287292	newly	16.	21.6441	{6., 42.}	73.1992	0.255475	Reff	0.619294	0.407597	{0.261581, 0.528007}	42.893	ar	671.	878.847	{845., 905.}	30.9758
11	R0	{1.60334, 19.9548}	{1.98725, 19.5716}	{{1.98971, 19.0665}, {1.98971, 19.6444}}	{23.9443, 1.92033}	inf	70.	64.551	{23., 118.}	39.2711	0.204487	newly	28.	21.0612	{7., 38.}	43.586	0.276877	Reff	0.80167	0.523821	{0.389553, 0.666553}	34.6588	ar	617.	799.061	{761., 834.}	29.5075
12	R0	{1.68623, 7.58585}	{2.81989, 15.1991}	{{2.66419, 14.2056}, {2.96947, 15.963}}	{67.2306, 100.361}	inf	7.	24.6716	{11., 40.}	252.452	0.483662	newly	7.	9.83582	{4., 17.}	63.113	0.213736	Reff	0.612101	0.228248	{0.16926, 0.309046}	62.7107	ar	640.	925.612	{902., 948.}	44.6269
13	R0	{1.82689, 18.0022}	{2.26366, 19.3888}	{{2.26161, 19.3849}, {2.26161, 19.3849}}	{23.9079, 7.70218}	inf	56.	66.2632	{27., 130.}	50.3446	0.18945	newly	15.	21.6316	{8., 43.}	69.9415	0.22862	Reff	0.668642	0.432825	{0.309841, 0.588019}	40.8266	ar	673.	860.947	{836., 884.}	27.9268
14	R0	{1.75911, 9.16883}	{2.89375, 19.0641}	{{2.8568, 18.4249}, {2.93978, 19.9817}}	{64.5007, 107.923}	inf	25.	76.6364	{45., 114.}	206.753	0.442518	newly	15.	24.5455	{11., 39.}	73.1602	0.208641	Reff	0.656148	0.267088	{0.173447, 0.350645}	60.0836	ar	661.	933.065	{912., 951.}	41.1596
15	R0	{1.46168, 11.3859}	{2.41419, 19.3838}	{{2.37462, 19.3966}, {2.42625, 19.5287}}	{65.1656, 70.2443}	inf	9.	32.2466	{11., 72.}	266.21	0.485157	newly	3.	10.3425	{3., 24.}	254.795	0.463552	Reff	0.654833	0.334701	{0.254084, 0.417315}	49.0489	ar	556.	878.507	{856., 903.}	58.0048
16	R0	{1.53577, 11.7581}	{2.74091, 18.1458}	{{2.56757, 16.884}, {2.93978, 19.9817}}	{78.4712, 54.3259}	inf	31.	61.2639	{31., 88.}	103.45	0.269755	newly	13.	20.7917	{9., 35.}	75.7479	0.227254	Reff	0.664988	0.28816	{0.219875, 0.367946}	56.667	ar	625.	915.042	{886., 935.}	46.4067
17	R0	{1.70698, 9.72924}	{2.83959, 16.4238}	{{2.78517, 15.7559}, {2.8939, 16.8013}}	{66.3516, 68.8089}	inf	8.	40.9324	{17., 76.}	411.655	0.634315	newly	4.	14.8919	{5., 25.}	273.649	0.484807	Reff	0.571838	0.238299	{0.18521, 0.30386}	58.3276	ar	675.	927.541	{909., 942.}	37.4134
18	R0	{1.5973, 14.931}	{1.9651, 12.7655}	{{1.83592, 11.3627}, {2.04669, 13.609}}	{23.0264, 14.5035}	inf	59.	55.7736	{19., 100.}	44.2277	0.223684	newly	28.	27.4528	{8., 56.}	48.3154	0.244521	Reff	0.967964	0.552619	{0.443687, 0.70539}	42.9092	ar	632.	777.019	{734., 812.}	22.946
19	R0	{1.87468, 17.004}	{2.26217, 19.3817}	{{2.26161, 19.3849}, {2.26161, 19.3849}}	{20.6698, 13.9832}	inf	48.	45.7636	{16., 88.}	53.447	0.250458	newly	12.	15.2727	{4., 35.}	71.5152	0.276108	Reff	0.684258	0.385283	{0.294009, 0.497554}	45.2612	ar	692.	860.855	{840., 884.}	24.4009
20	R0	{1.84715, 12.531}	{2.2732, 19.3306}	{{2.26161, 19.0355}, {2.29738, 19.3849}}	{23.0655, 54.2621}	inf	9.	50.8136	{18., 95.}	466.478	0.675294	newly	3.	16.7119	{5., 33.}	458.192	0.650059	Reff	0.676057	0.400498	{0.291748, 0.53374}	43.483	ar	661.	859.831	{836., 884.}	30.0803
21	R0	{1.75938, 13.5959}	{2.62178, 17.2409}	{{2.46246, 16.0321}, {2.76849, 18.33}}	{49.0172, 26.8096}	inf	38.	46.2419	{18., 77.}	55.3056	0.199337	newly	12.	16.4839	{6., 31.}	71.2366	0.253183	Reff	0.777646	0.29412	{0.222545, 0.402267}	62.1782	ar	620.	904.323	{874., 927.}	45.8585
22	R0	{1.63456, 10.7469}	{2.41679, 19.4378}	{{2.42205, 19.4475}, {2.42205, 19.4475}}	{47.856, 80.8692}	inf	11.	53.1333	{18., 98.}	383.636	0.576118	newly	7.	17.0333	{5., 33.}	158.571	0.342228	Reff	0.568827	0.383275	{0.268848, 0.474722}	39.8933	ar	654.	878.283	{845., 903.}	34.2941
23	R0	{1.62702, 19.1392}	{2.40606, 19.3721}	{{2.37462, 19.391}, {2.42625, 19.5287}}	{47.8817, 1.98524}	inf	42.	62.6522	{24., 123.}	70.4279	0.217993	newly	16.	20.3913	{8., 44.}	60.5978	0.216409	Reff	0.715889	0.388623	{0.276114, 0.477971}	49.0131	ar	600.	878.855	{851., 903.}	46.4758
24	R0	{1.70466, 12.1202}	{2.27289, 18.7665}	{{2.23915, 18.4504}, {2.37462, 19.3966}}	{33.3342, 54.8363}	inf	15.	31.2586	{9., 62.}	126.092	0.285993	newly	6.	10.2931	{2., 22.}	110.632	0.307938	Reff	0.685273	0.385231	{0.283871, 0.479178}	45.3206	ar	613.	851.586	{815., 887.}	38.9211
25	R0	{1.61735, 11.3169}	{2.12601, 19.335}	{{2.11476, 19.2116}, {2.12588, 19.5406}}	{31.4501, 70.8507}	inf	17.	44.129	{16., 75.}	166.793	0.356839	newly	11.	14.3226	{5., 29.}	71.261	0.257086	Reff	0.533726	0.454006	{0.332017, 0.522346}	30.8513	ar	681.	835.	{800., 869.}	22.6138
26	R0	{1.56977, 12.3035}	{2.55152, 17.7207}	{{2.30108, 15.4658}, {2.78218, 19.8588}}	{62.5409, 44.0301}	inf	23.	33.7059	{15., 72.}	71.6113	0.231739	newly	15.	11.4412	{4., 21.}	46.2745	0.285872	Reff	0.758199	0.288289	{0.216078, 0.387884}	61.9771	ar	594.	898.088	{854., 930.}	51.1933
27	R0	{1.46317, 5.8298}	{2.60937, 18.4974}	{{2.46976, 17.5057}, {2.78218, 19.8588}}	{78.3364, 217.29}	inf	0.001	52.2206	{18., 98.}	5.22196×10^{6}	2.62469	newly	0.1	17.1471	{6., 34.}	17047.1	2.11254	Reff	0.605752	0.305816	{0.205801, 0.392692}	50.6627	ar	586.	905.191	{876., 932.}	54.4695
28	R0	{1.70002, 14.9806}	{2.79406, 19.7694}	{{2.76795, 19.6041}, {2.85956, 19.984}}	{64.3547, 31.9664}	inf	50.	81.9254	{38., 126.}	71.9701	0.207826	newly	31.	25.791	{12., 44.}	40.6837	0.213595	Reff	0.683408	0.295398	{0.213132, 0.430902}	57.0162	ar	652.	922.851	{899., 940.}	41.5415
29	R0	{1.52729, 16.1014}	{2.26661, 19.3899}	{{2.26161, 19.3211}, {2.27287, 19.5128}}	{48.4073, 20.4235}	inf	25.	31.0862	{9., 71.}	74.4138	0.26824	newly	10.	9.51724	{3., 27.}	72.7586	0.372353	Reff	0.720881	0.363021	{0.275916, 0.490769}	49.8071	ar	592.	861.362	{836., 887.}	45.5003
30	R0	{1.72795, 13.5635}	{2.42114, 19.4456}	{{2.42205, 19.4475}, {2.42205, 19.4475}}	{40.1165, 43.3672}	inf	17.	43.5085	{14., 88.}	165.304	0.330916	newly	2.	14.6102	{3., 31.}	630.508	0.719696	Reff	0.60651	0.362198	{0.254315, 0.465034}	44.3452	ar	668.	879.136	{845., 905.}	31.6071
31	R0	{1.51591, 18.1544}	{2.16511, 18.6133}	{{1.98261, 16.748}, {2.26107, 19.5128}}	{42.8255, 6.10683}	inf	38.	38.0161	{14., 66.}	43.5059	0.195994	newly	24.	12.6613	{4., 24.}	54.6371	0.404335	Reff	0.858005	0.421235	{0.319594, 0.514051}	52.6749	ar	541.	834.532	{799., 871.}	54.2573
32	R0	{1.4282, 10.3534}	{2.17227, 19.2725}	{{2.12588, 19.1478}, {2.26107, 19.9579}}	{52.0986, 86.1462}	inf	37.	107.738	{57., 160.}	191.449	0.433682	newly	27.	36.6885	{20., 59.}	50.8197	0.178132	Reff	0.854064	0.606295	{0.40247, 0.950268}	36.7329	ar	521.	833.246	{797., 870.}	59.932
33	R0	{1.56693, 11.3972}	{2.91084, 18.8936}	{{2.8568, 18.4249}, {2.97157, 19.3547}}	{85.7668, 65.7743}	inf	21.	73.1127	{36., 121.}	248.156	0.487729	newly	13.	23.3803	{12., 36.}	87.4323	0.243588	Reff	0.518654	0.268357	{0.188549, 0.343381}	48.8493	ar	695.	931.408	{915., 948.}	34.0156
34	R0	{1.50675, 19.8823}	{2.2616, 19.2176}	{{2.23915, 18.4504}, {2.27287, 19.5128}}	{50.098, 3.34323}	inf	31.	35.4127	{11., 78.}	65.4378	0.258682	newly	10.	11.3333	{2., 24.}	70.7937	0.29965	Reff	0.729267	0.386596	{0.297179, 0.4772}	48.126	ar	587.	854.317	{823., 883.}	45.5396
35	R0	{1.72889, 7.76068}	{2.97457, 18.569}	{{2.96797, 18.1089}, {2.99088, 18.9406}}	{72.0507, 139.27}	inf	3.	63.6164	{31., 117.}	2020.55	1.27558	newly	3.	20.2466	{10., 37.}	574.886	0.775521	Reff	0.605112	0.243885	{0.179453, 0.30529}	59.6958	ar	650.	935.342	{921., 947.}	43.8988
36	R0	{1.62644, 4.12303}	{2.85667, 11.8905}	{{2.80569, 11.6897}, {2.96796, 12.4643}}	{75.6392, 188.392}	inf	0.001	7.63889	{2., 16.}	763792.	1.7326	newly	0.1	3.63889	{1., 9.}	3552.78	1.3665	Reff	0.566001	0.21612	{0.165536, 0.269346}	61.8164	ar	652.	926.361	{907., 944.}	42.0799
37	R0	{1.70878, 15.8072}	{2.15839, 19.5582}	{{2.12588, 19.2116}, {2.1968, 19.9579}}	{26.3119, 23.7296}	inf	40.	33.5667	{12., 59.}	53.	0.276569	newly	11.	10.8833	{3., 22.}	59.2424	0.275814	Reff	0.659589	0.431962	{0.306127, 0.553594}	41.6706	ar	674.	836.05	{794., 872.}	24.043
38	R0	{1.58967, 6.80568}	{2.86236, 17.1161}	{{2.60786, 15.5255}, {2.98558, 18.1089}}	{80.06, 151.497}	inf	5.	32.7727	{15., 57.}	555.455	0.758766	newly	3.	11.4242	{5., 21.}	284.848	0.525747	Reff	0.62474	0.229424	{0.179135, 0.292587}	63.277	ar	610.	928.97	{906., 947.}	52.2901
39	R0	{1.344, 8.19498}	{2.28496, 19.1487}	{{2.25962, 18.788}, {2.30371, 19.6625}}	{70.0116, 133.663}	inf	14.	63.1045	{30., 97.}	350.746	0.604827	newly	14.	20.8955	{8., 36.}	67.3774	0.210863	Reff	0.684096	0.428497	{0.33312, 0.539884}	38.5127	ar	551.	855.91	{824., 886.}	55.3376
40	R0	{1.61509, 7.90825}	{2.49803, 12.3639}	{{2.32891, 10.9956}, {2.7346, 14.003}}	{54.668, 56.342}	inf	13.	18.9028	{5., 38.}	79.594	0.26738	newly	9.	7.97222	{2., 16.}	55.8642	0.308849	Reff	0.7171	0.286942	{0.192187, 0.36801}	59.9858	ar	581.	891.056	{849., 935.}	53.3658
41	R0	{1.60017, 5.90605}	{2.91134, 19.0648}	{{2.8568, 18.4275}, {2.93978, 19.9817}}	{81.9395, 222.8}	inf	6.	48.6533	{19., 89.}	710.889	0.833211	newly	6.	14.8	{5., 26.}	152.444	0.338479	Reff	0.547258	0.240057	{0.171994, 0.317496}	56.1345	ar	661.	931.587	{913., 950.}	40.936
42	R0	{1.64436, 15.1337}	{2.40931, 17.7314}	{{2.24242, 15.7579}, {2.53519, 19.5955}}	{46.5195, 17.1715}	inf	40.	55.2319	{24., 107.}	57.6449	0.187999	newly	15.	19.2174	{8., 35.}	57.1014	0.19924	Reff	0.779427	0.379372	{0.263884, 0.466423}	52.201	ar	640.	872.899	{834., 910.}	36.3904
43	R0	{1.48917, 11.917}	{2.12762, 19.5172}	{{2.11476, 19.2116}, {2.1968, 19.9579}}	{42.8728, 63.7763}	inf	13.	42.4603	{16., 69.}	229.304	0.441368	newly	9.	15.0317	{5., 28.}	95.2381	0.287334	Reff	0.775858	0.457782	{0.323558, 0.583674}	46.9423	ar	551.	829.46	{794., 866.}	50.5373
44	R0	{1.65577, 7.89317}	{2.76184, 18.8739}	{{2.56757, 16.884}, {2.93978, 19.9817}}	{66.801, 139.117}	inf	7.	56.7763	{23., 105.}	711.09	0.837756	newly	5.	18.1053	{6., 38.}	262.632	0.46223	Reff	0.738473	0.280557	{0.179327, 0.358762}	64.7134	ar	576.	922.	{890., 945.}	60.0694
45	R0	{1.6909, 12.3275}	{2.57573, 19.4934}	{{2.50427, 19.1373}, {2.66225, 19.9254}}	{52.329, 58.1296}	inf	23.	54.8923	{25., 92.}	140.669	0.324347	newly	8.	18.0615	{7., 33.}	131.154	0.309571	Reff	0.654378	0.327634	{0.23694, 0.423298}	50.6231	ar	675.	897.185	{872., 920.}	32.9162
46	R0	{1.62351, 16.0698}	{2.10401, 19.3973}	{{1.99771, 19.0665}, {2.12588, 19.6444}}	{29.5961, 20.7067}	inf	51.	59.7419	{28., 98.}	46.8058	0.19126	newly	13.	19.0484	{8., 33.}	69.1067	0.230382	Reff	0.899425	0.4922	{0.361624, 0.630198}	49.2326	ar	591.	824.548	{785., 857.}	39.5175
47	R0	{1.52372, 4.38599}	{2.83141, 14.2371}	{{2.73634, 13.3345}, {2.86193, 14.6465}}	{85.8219, 224.603}	inf	0.001	25.7667	{7., 48.}	2.57657×10^{6}	2.27004	newly	0.1	10.5	{2., 19.}	10403.3	1.84389	Reff	0.582061	0.217305	{0.168854, 0.271883}	62.6663	ar	618.	930.467	{916., 947.}	50.5609
48	R0	{1.44306, 11.788}	{2.41487, 18.463}	{{2.24606, 16.668}, {2.49423, 19.3027}}	{67.3435, 56.625}	inf	22.	29.1406	{13., 54.}	56.321	0.195548	newly	12.	10.3281	{4., 19.}	45.4427	0.269701	Reff	0.722973	0.337806	{0.247923, 0.453704}	53.2754	ar	534.	873.484	{843., 903.}	63.5739
49	R0	{1.63624, 11.3893}	{2.90585, 18.611}	{{2.8568, 18.2127}, {2.97157, 18.9406}}	{77.5931, 63.408}	inf	22.	41.9726	{14., 62.}	103.736	0.2519	newly	9.	13.5342	{4., 20.}	79.9087	0.245968	Reff	0.623407	0.235863	{0.166171, 0.309421}	62.2758	ar	643.	931.288	{907., 948.}	44.8348
50	R0	{1.46113, 11.1322}	{2.25273, 18.196}	{{2.19504, 17.1709}, {2.30371, 19.3966}}	{54.1775, 63.4542}	inf	28.	68.7612	{30., 122.}	147.281	0.348228	newly	17.	23.3881	{10., 43.}	55.8385	0.184565	Reff	0.831383	0.449786	{0.325118, 0.653543}	46.9868	ar	501.	853.478	{820., 887.}	70.3548
